# Diagnosis and Treatment of Status Epilepticus in a Pediatric Renal Recipient

**DOI:** 10.1155/2011/706107

**Published:** 2012-01-05

**Authors:** Gao Hongjun, Luo Xiangdong, Liang Taisheng, Lu Shangguang, Liang FangFang, Dong Yu, Tan Zhen, Wu Zhen

**Affiliations:** RuiKang Hospital, Affiliated to GuangXi Chinese Traditional Medicine College, Nanning 530011, China

## Abstract

We elaborate on the retrospective analysis of clinical data on a patient afflicted with grand mal seizures following a kidney transplant. The 16-year-old female patient was hospitalized for chronic glomerulonephritis. She experienced an epileptic seizure and was treated with carbamazepine. Renal transplantation was performed; the function of the transplant kidney was normal. However, grand mal seizures, which required intravenous and luminal intramuscular diazepam injections for control, began on the fourth postoperative day and lasted for 3 days, occurring approximately 10 to 20 times per day. On the sixth day, the patient fell into a deep comatose state and developed the inability to move the right side of her body, hypomyotonia, type 1 respiratory failure, and a pulmonary infection. She was given a breathing machine to assist with respiration. At the same time, she was given protection from infection, tranquilization, treatment for dehydration and diuresis, supportive therapy for the right side of her body, and adjustment of her immunosuppressants. On the 12th postoperative day, the patient's consciousness gradually returned; on the 15th day, the breathing machine was removed with recovery of myodynamia; on the 27th day, she was fully cured with no neurological sequelae.

## 1. Introduction

Under the effect of immunosuppression and other factors, renal transplant recipients are likely to be afflicted with epilepsy, the attack rate of which can reach almost 20% among pediatric patients. However, at present, there are insufficient statistics to explain the cause of this danger [1]. Status epilepticus, also called grand mal seizures, can have a serious effect on the prognosis of renal transplant recipients. In particular, the first few attacks after kidney transplantation may have a serious effect on renal transplant recipients.

## 2. Briefing of Case History

### 2.1. Object

A 17-year-old female patient was hospitalized because of dizziness and fatigue of more than 2 months' duration and with no obvious precipitating factor. Without timely treatment, she soon experienced syncope. She was hospitalized and examined, judged to have high blood pressure and a serum creatinine (SCr) level of 1374.9 *μ*mmol/L, diagnosed with chronic glomerulonephritis and uremia, and treated with hemodialysis.

### 2.2. Preoperative Diagnosis and Treatment

After being hospitalized, the patient was treated with regular hemodialysis and underwent a series of preoperative therapeutic measures including blood pressure control, anemia improvement, and weight control. On the 40th preoperative day, the patient developed acute loss of consciousness, expressionless eyes, foaming at the mouth, and convulsion of the limbs, all of which lasted for about 5 min. Cerebral MRI was performed, but no abnormalities were detected. The patient was then diagnosed with symptomatic epilepsy. Carbamazepine was initially administered at a dosage of 0.1 g twice a day; the concentration was checked at regular intervals to adjust the dosage. After 2 weeks, the patient was treated with maintenance therapy at a dosage of 0.1 g once a day. This therapy was successful, and the patient was never again afflicted with epilepsy. The patient confirmed that there had been no record of epilepsy in her previous case history.

### 2.3. Surgery

Renal transplantation was performed. The transplanted kidney secreted urine normally during establishment of its blood supply, but the postoperative urine amount gradually decreased until it reached a low of only 368 mL at the 14th postoperative hour. Ten hours postoperatively, reversed blood flow was observed in the transplant renal vein by color Doppler ultrasound, which suggested the presence of renal vein thrombosis (RVT). Thus, removal of the RVT was immediately implemented. After the operation, the patient was treated with urokinase thrombolysis and heparin anticoagulant therapy. As a result, transplant renal function rapidly returned to normal with urine volume maintaining a level of 4000 to 5000 mL/d and no worsening of the creatinine level. The patient was not disturbed and experienced no discomfort.

### 2.4. The Patient's Postoperative Condition

#### 2.4.1. Conditions on Postoperative Days 3 and 4

During these 2 days, the general condition of the patient was well, and she was able to perform the Q & A. With the exception of pain associated with the surgical incision, the patient reported no discomfort. Urine volume was normal, being maintained at approximately 5000 mL/d. The heparin anticoagulant therapy was performed with a continuous infusion of liquaemin for 24 h. On the fourth postoperative day, anticoagulation therapy was stopped on the basis of PT and APTT levels, and conventional postoperative antirejection therapy was implemented.

#### 2.4.2. Conditions on Postoperative Days 5 to 11

**Figure 1:**
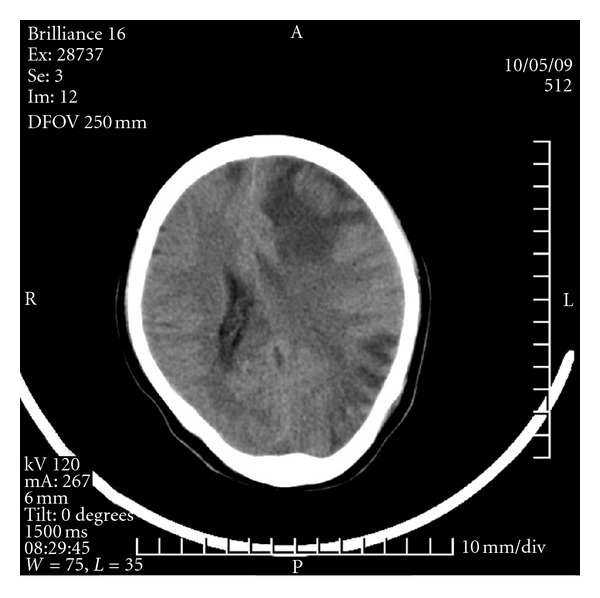
The frist day CT scan.

**Figure 2:**
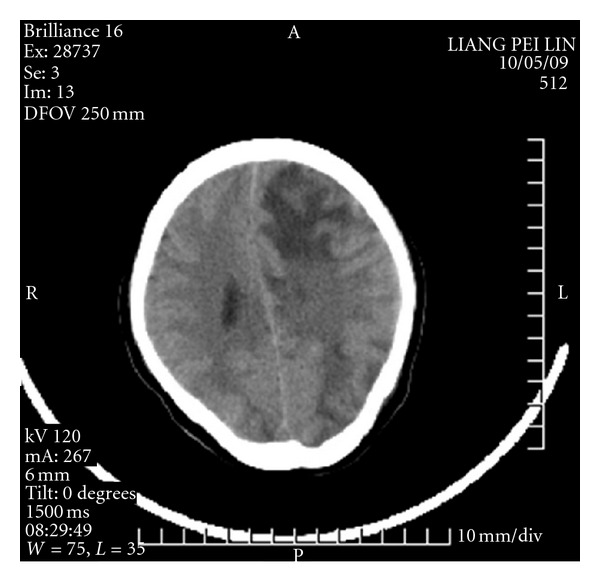
The frist day CT scan.

**Figure 3:**
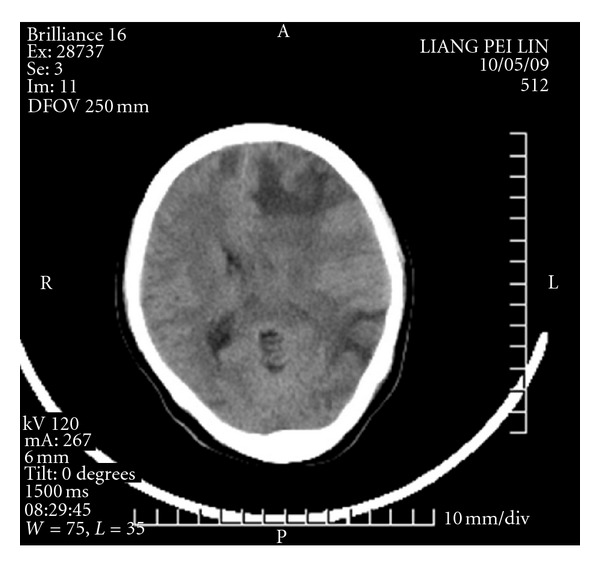
The frist day CT scan.

At 4:30 a.m. on the fifth day, the patient experienced acute paroxysmal convulsions of her lower right extremity with no obvious precipitating factor; the convulsions subsequently spread to both upper and lower extremities. Her entire body was afflicted with persistent tonic convulsions, and she experienced loss of consciousness, expressionless eyes, frothing at the mouth, and gatism, all of which lasted approximately 2 min. The patient was given a pressure pad to protect her tongue and was treated with intravenous and luminal intramuscular injections of diazepam and oral carbamazepine, which had no obvious effect. The patient experienced the aforementioned typical signs of epilepsy at intervals ranging from 30 min to 2 h. These typical epileptic seizures occurred 10 times in 24 h. Given the frequency of status epilepticus and the unusually severe drug effect, the patient underwent a neurological consultation and was treated with a continuous intravenous drip of 500 mL 5% glucose plus 100 mg diazepam at 10 mL/h. The patient fell into somnolence, and her symptoms improved. During treatment with diazepam, although no typical epileptic attacks occurred, atypical epileptic seizures occurred 10 times in the form of twitching of the corners of the mouth and right upper extremity. On the sixth postoperative day, there were still no typical epileptic attacks with continuous use of diazepam. With a decreasing dose of diazepam, the patient was in a superficial coma, afflicted with hypomyotonia, and had an inability to move the right side of her body. A head CT scan revealed a nodule with an unclear outline in the left frontal lobe, around which patches of edema were observed. Patches of edema were also observed in the right semioval center, and the left tricorn was narrowed by the pressure. The midline shifted toward the left, resulting in edema of the brain tissue (Figures 1, 2, and 3).

The patient's tranquilization was continued, and she was treated for dehydration and diuresis and given supportive therapy for the right side of her body. From the seventh postoperative day, the patient was in a lighter comatose state but was afflicted with type 1 respiratory failure and pulmonary infection. Thus, she was given a breathing machine and anti-infective treatment.

#### 2.4.3. Conditions on Postoperative Days 12 to 30

**Figure 4:**
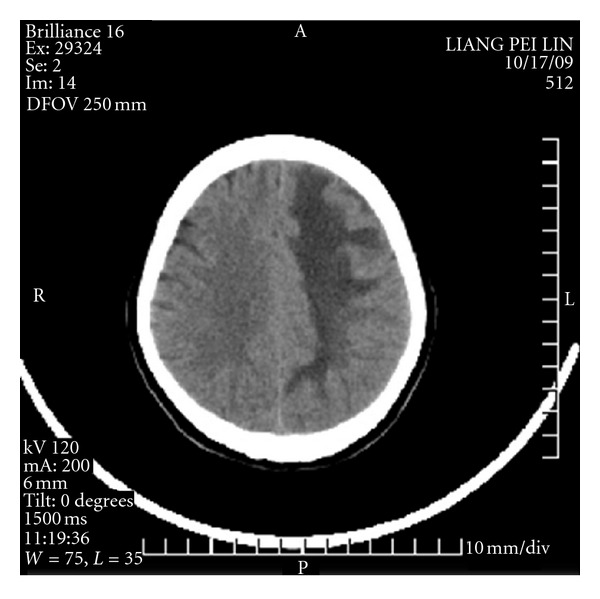
The 12th day CT scan.

**Figure 5:**
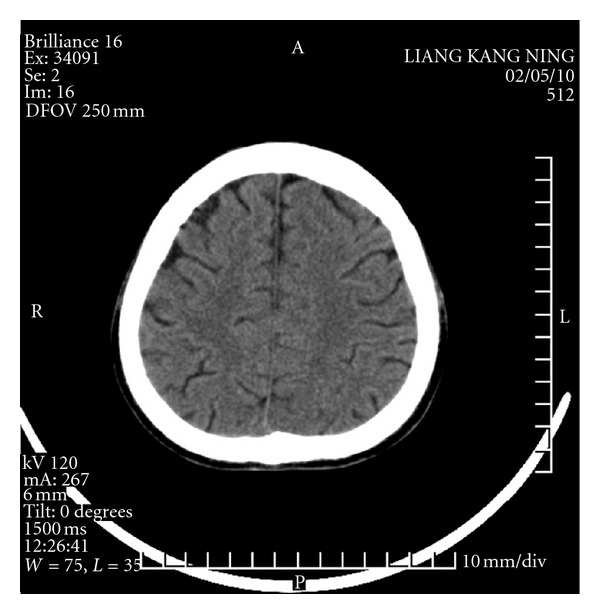
The 30th day CT scan.

On the 12th postoperative day, the patient's condition improved with a gradual recovery of consciousness and myodynamia of the extremities. The muscular tension level of the left extremities was low, while that of the right was zero. On the 15th postoperative day, the breathing machine was removed; the muscular tension level of the left extremities returned to normal, and that of the right was five. Head CT revealed large patches of low-density areas in the semioval center of the left frontal lobe, and the left ventricle was under a small amount of pressure (Figures 4 and 5). The patient was able to perform early ambulation, but discordance of the right extremities was still observed. Head CT on the 27th postoperative day revealed the persistence of irregular low-density areas in the left frontal lobe, and the left frontal angle had moved back slightly. With normal movement of the extremities, an almost-normal muscular tension level of the left extremities, and elimination of the pulmonary infection, the patient was cured and discharged from the hospital.

#### 2.4.4. Followups from Hospital Discharge to Present

At present, the general health condition, spirit, and appetite are good; the transplant kidney functions well; no neurological sequelae remain; no abnormalities exist on head CT.

### 2.5. The Application of Immunosuppressive Agents

During the operation, 350 mg methylprednisolone was administered *via* intravenous injection, and in the following 3 days, the use of methylprednisolone was continued at a dosage of 350 mg in the first 2 days and 200 mg on the third day. tacrolimus (FK506) plus mycophenolate mofetil (MMF) plus prednisone was implemented postoperatively. Oral administration of FK506 at a dosage of 5 to 7 mg/kg/d and MMF at a dosage of 0.5 mg twice daily was begun one day preoperatively. Because of carbamazepine's effect on FK506, the amount of FK506 was increased to achieve the target concentration.

## 3. Discussion

Status epilepticus is a pathological condition characterized by continuous and frequent seizures and is classified as a neurological emergency. According to conventional standards, status epilepticus consists of attacks lasting more than 30 min or repeated attacks during which consciousness is lost. New diagnostic criteria have recently been proposed by Lowenstein and other experts [1], and among adults and children older than 5 years of age, generalized convulsive status epilepticus refers to a continuous seizure of more than 5 minutes in duration or more than 2 seizures at a time; during the seizure, the patient's consciousness is lost. A common clinical, and the most dangerous, form is the persistent state of the generalized tonic-clonic seizure. Symptoms are a sudden loss of consciousness, muscle twitching, foaming (sometimes with blood) at the mouth, frequent apnea, cyanosis, dilated pupils, a diminishing papillary light response, and gatism. The seizure is long in duration or occurs repeatedly. If not quickly controlled, it may be life threatening or cause perpetual brain damage. Status epilepticus is mainly caused by improper drug decrease and withdrawal, sudden changes in medication, or nonstandard times of antiepileptic treatment administration; it may also be induced by infection, mental factors, fatigue, pregnancy, drinking, and other causes. Infection, birth injury, and congenital malformation are the main causes in infancy and childhood. Common causes in young adults include traumatic brain injury, intracranial masses, and parasitic diseases, while stroke, brain tumors, trauma, and degenerative diseases are the main causes in the elderly.

Neural complications in renal transplant recipients are likely to occur at any stage of the postoperative period and have an incidence of 30% to 60%. Both the neural complication incidence and the mortality have a great effect on renal transplant recipients [2–4]. The characteristics of neural complications caused by renal transplants are distinct from those caused by other organ transplants and include limb tremors, insomnia, dysphoria, coma, and convulsions. Epilepsy also occurs among renal transplant recipients [5], and the incidences among adults and children are 11.4% and 17.6%, respectively [6]. In cats with end-stage renal failure that have undergone renal transplants, the incidence of epilepsy may reach 28.9% [7].

Many factors can lead to epilepsy after transplantation, among which electrolyte disturbance is the most common [8]. Characteristics include early epileptic attacks, usually in the 72nd postoperative hour, a high urine volume at 10 000 mL/d, rapid recovery of renal function, and the return of SCr to a normal level at the first epileptic attack. Epilepsy can be easily controlled by correction of electrolyte disturbances with supplementation of blood calcium, magnesium, and sodium and intramuscular injections of diazepam. However, the causes of status epilepticus might be linked to withdrawal of antiepileptic drugs (AEDs) and intoxication [9] of the calcineurin inhibitors cyclosporine and tacrolimus. Epilepsy can be caused by intoxication of cyclosporine and tacrolimus [10], the incidences of which are 2% to 6% [11–14] and 5.6% to 11.6% [15]. Antirejection therapy can be impacted by the use of larger doses of hormones, by which the patient's mental state can be changed and epilepsy can be easily triggered. This type of epilepsy has more severe symptoms, easily causes acute respiratory distress syndrome, and maintains a high mortality; it is also very difficult to cure, often requiring tracheal cannulation and positive end-expiratory pressure with a breathing machine. The aforementioned patient was afflicted by epilepsy once during the month before the transplant. Because of AED withdrawal, the impact of MMP, and the use of tacrolimus, children and young adults with a history of epilepsy are afflicted with epilepsy more frequently, and its persistent state is caused by neurological diseases under the combined action of hormones and physiological changes. It was found by Gleeson through 3-year followups of patients with epileptic seizures during the perioperative period during AED withdrawal that the patients were never afflicted with epilepsy [16].

The grand mal seizures after renal transplantation were usually serious. However, the patient was cured without neurological sequelae, and the transplant kidney recovered very well. Experience with this diagnosis and treatment regimen has been gained in the following 4 respects. First, while the patient's life must be saved, transplant renal function must be maintained, and delayed graft function (DGF) must be prevented, which is the core of successful treatment. Changes in the patient's blood pressure and heart rate can be caused by epileptic seizures. In particular, status epilepticus has a more serious effect on the circulatory system, easily triggering DGF because of the adverse effect on transplant renal function. Once DGF has been triggered, the whole treatment will be more difficult. Second, accurate judgment of the causes of epilepsy is critical to effective diagnosis and treatment. Different causes, such as brain tumors, cerebrovascular accident, and drug-induced factors, require different treatments. However, the treatment should be simplified in case the patient's condition is complicated. Third, when epilepsy occurs during the perioperative period, immunosuppressive agents [17, 18] should be appropriately adjusted. Because of the interaction between AEDs and antirejection drugs, blood concentrations of cyclosporine and tacrolimus can be dramatically lowered by the carbamazepine. Thus, blood concentrations of carbamazepine and tacrolimus should be monitored so that the adjustment of immunosuppressive agents can be performed in time. Finally, comprehensive measures should be taken to prevent other complications and reduce adverse effects in the therapeutic process. Aspiration can easily be caused by seizures, and status epilepticus requires patients to stay in bed; both increase the possibility of pulmonary infection. In addition, central respiratory problems can be caused by severe cerebral problems. The importance of prevention and cure of pulmonary infection is determined by the complexity, refractory characteristics, and high mortality of pulmonary infection after transplant.

The improvement of surgical techniques of organ transplantation and specific nursing practices that can reduce mortality and morbidity make it possible to perform many organ transplantations. The postoperative period of organ transplantation mostly focuses on infection prevention and antirejection treatment. Most neural complications are atypical and have not been given enough attention. However, a certain amount of morbidity and mortality is still attributed to serious neural complications. On one hand, neural complications in some renal transplant recipients are caused by postoperative diseases. On the other hand, neurotoxicity caused by immunotherapy medication can also lead to a series of complications. The adverse effects of the immune overreaction to cyclosporine and tacrolimus should be noted [17]. Many factors, acting alone or in combination, can cause epileptic seizures after renal transplantation. Thus, for prevention and cure, great importance should be attached to comprehensive treatment. Drugs that can trigger epileptic seizures should be avoided. If epileptic seizures take place, the pathogenesis should be determined and symptomatic therapy begun.
